# A dosimetric comparison of 3D conformal vs intensity modulated vs volumetric arc radiation therapy for muscle invasive bladder cancer

**DOI:** 10.1186/1748-717X-7-111

**Published:** 2012-07-23

**Authors:** Farshad Foroudi, Lesley Wilson, Mathias Bressel, Annette Haworth, Colin Hornby, Daniel Pham, Jim Cramb, Suki Gill, Keen Hun Tai, Tomas Kron

**Affiliations:** 1 Division of Radiation Oncology, Peter MacCallum Cancer Center, Peter MacCallum Cancer Institute, St Andrews Place, East Melbourne, VIC, 3002, Australia; 2 Radiation Therapy Services, Peter MacCallum Cancer Center, Melbourne, VIC, Australia; 3 Biostatistics and Clinical Trials, Peter MacCallum Cancer Centre, Melbourne, VIC, Australia; 4 Physical Sciences, Peter MacCallum Cancer Center, Melbourne, VIC, Australia

**Keywords:** Bladder cancer, Intensity modulated radiation therapy, Volumetric modulated arc therapy

## Abstract

**Background:**

To compare 3 Dimensional Conformal radiotherapy (3D-CRT) with Intensity Modulated Radiotherapy (IMRT) with Volumetric-Modulated Arc Therapy (VMAT) for bladder cancer.

**Methods:**

Radiotherapy plans for 15 patients with T2-T4N0M0 bladder cancer were prospectively developed for 3-DCRT, IMRT and VMAT using Varian Eclipse planning system. The same radiation therapist carried out all planning and the same clinical dosimetric constraints were used. 10 of the patients with well localised tumours had a simultaneous infield boost (SIB) of the primary tumour planned for both IMRT and VMAT. Tumour control probabilities and normal tissue complication probabilities were calculated.

**Results:**

Mean planning time for 3D-CRT, IMRT and VMAT was 30.0, 49.3, and 141.0 minutes respectively. The mean PTV conformity (CI) index for 3D-CRT was 1.32, for IMRT 1.05, and for VMAT 1.05. The PTV Homogeneity (HI) index was 0.080 for 3D-CRT, 0.073 for IMRT and 0.086 for VMAT. Tumour control and normal tissue complication probabilities were similar for 3D-CRT, IMRT and VMAT. The mean monitor units were 267 (range 250–293) for 3D-CRT; 824 (range 641–1083) for IMRT; and 403 (range 333–489) for VMAT (P < 0.05). Average treatment delivery time were 2:25min (range 2:01–3:09) for 3D-CRT; 4:39 (range 3:41–6:40) for IMRT; and 1:14 (range 1:13–1:14) for VMAT. In selected patients, the SIB did not result in a higher dose to small bowel or rectum.

**Conclusions:**

VMAT is associated with similar dosimetric advantages as IMRT over 3D-CRT for muscle invasive bladder cancer. VMAT is associated with faster delivery times and less number of mean monitor units than IMRT. SIB is feasible in selected patients with localized tumours.

## Introduction

Three-dimensional conformal radiotherapy (3D-CRT) has historically been the standard modality for external beam radiotherapy (RT) for bladder cancer. The morbidity of bladder cancer treated with conventional radiotherapy is well known with a RTOG update showing that 7% of their patients experienced late grade 3+ pelvic toxicity [1]. Intensity modulated radiation therapy (IMRT) can treat less of the surrounding normal tissues potentially reducing normal tissue side effects. [Hsieh et al. 2] found that IMRT provided good locoregional progression free survival particularly in the elderly bladder cancer group. Another advantage of IMRT is the ability to deliver more than one dose level to target volumes at the same time. This provides for the potential to deliver a simultaneous in-field boost to well localised primary sites of the tumor [3,4]. However, the potential negative of IMRT include the increased time required for RT delivery and the associated risk of bladder filling and changes in bladder shape and size. The magnitude of bladder filling during treatment delivery has recently been demonstrated to be approximately 1cm^3^ per minute, but with wide inter patient variation [5]. It would be particularly problematic if highly conformal dose distributions are used which are typical for IMRT. Another disadvantage of IMRT is the increased number of monitor units (MU) needed, which results in a greater integral body dose, with a probable increased risk of second malignancies [6].

VMAT is a novel type of IMRT which may be able to address both the shortcomings of IMRT discussed above. In VMAT, gantry speed, multileaf collimator (MLC) leaf position and dose rate are dynamically varied during rotation of the gantry yielding a fast and highly conformal treatment delivery [7]. While many reports on the clinical use of VMAT in the context of prostate, cervix and head and neck cancer exist [8-12] there is to our knowledge no report on the suitability of VMAT for radiotherapy of bladder cancer.

RapidArc (*Varian Medical Systems, Palo Alto, CA*) utilises the optimisation algorithm first described by [Otto, K 13] to plan VMAT. This technology has recently been implemented clinically and is becoming more widely available in radiotherapy centres. Planning studies comparing VMAT with IMRT for the primary treatment of prostate cancer have demonstrated some improvements in plan quality [14,15]. Compared with IMRT, the potential advantages of VMAT include a large reduction in the number of MUs, with an associated reduction in treatment time, required to deliver a given fraction size.

We sought to determine if the dosimetric parameters for 3D-CRT, IMRT and VMAT for muscle invasive bladder cancer can be similar. If dosimetric outcome was similar we aimed to compare monitor units (MU) and required beam delivery time as well as planning time required for three modalities. We also aimed to compare normal tissue complication probability and tumour control probabilities between the modalities. Finally in patients with localised tumours amenable to a boost we examined the feasibility of a simultaneous infield boost (SIB) of the primary tumour for the IMRT and VMAT techniques.

## Method

All 15 participant images were from a human ethics approved bladder cancer protocol, full details of this protocol have been previously published [16]. Patients underwent CT simulation using 3mm thick slices by 3mm spacing, after emptying their bladder completely. Immobilisation was with ankle stocks and a bolster under the knees. A single radiation oncologist (FF) completed contouring of the bladder, primary tumour, rectum, and non-rectal bowel prior to commencement of the project. All 3D-CRT, IMRT and VMAT plans were created prospectively using the unique set of contours for each patient by one experienced radiation therapist investigator (LW). A standalone Eclipse (Varian, Palo Alto, CA) treatment planning system, installed on a Dell Precision T5500 computer, was used for all plan creation. The radiation therapist had extensive experience with 3D-CRT and IMRT planning, as well as training and although lesser experienced with VMAT planning was supported by a very experienced VMAT planner to mitigate any bias in recorded planning times.

The treatment plans were based on whole bladder treatment with a single phase treatment. The study was based on the original planning CT and contoured volumes in each of the 15 patients. The CTV consisted of the gross tumour volume (GTV) and the whole bladder. As outlined in Australian and New Zealand consensus guidelines for bladder cancer radiotherapy [17], pelvic lymph nodes were not an elective part of the volume in any patient. The CTV to PTV margin was 1.5cm. No oral or intravenous contrast was used at simulation. The rectum and non rectal bowel (all bowel not considered part of the rectum) were contoured 4 slices (12mm) above and below the PTV. Each patient had one 3D CRT, one IMRT and one VMAT plan created by the same radiation therapist (LW). The same dose constraints were used for creation of 3D CRT, IMRT and VMAT plans (Table 1). 

**Table 1:** Clinical Dosimetric Constraints used in Planning

	**3D-CRT/IMRT/VMAT**
PTV	D2% ≥ 60.8Gy
	D98% ≤ 68.5Gy
	Median absorbed dose (D50%) to be ±2% of 64Gy in 32 fractions of 2Gy each
Bladder	V15 ≤ 80%
	V25 ≤ 75%
	V35 ≤ 70%
	V50 ≤ 65%
Rectum	D50 ≤ 50Gy
	D40 ≤ 64Gy
	V50 < 50%,
	V60 < 35%,
	V65 < 25%,
	V70 < 20%,
	V75 <15%
Rt Fem Head and Lt Fem Head	D100 ≤ 35Gy
	D60 ≤ 45Gy
	D10 ≤ 50Gy
Small bowel (non rectal)	V15Gy ≤ 350cc
	V30Gy ≤ 150cc
	V35Gy ≤ 120cc
	V45Gy ≤ 50 cc
	V50Gy = 0 cc

The total prescription dose to the PTV was based on a median dose of 64Gy delivered in 32 fractions at 2Gy per fraction treating daily, five days per week over six and the half weeks. Plans were normalised to ensure that the 95% isodose adequately covered the PTV and that the dose distribution was such that the minimum dose to 99% of the PTV (*D*_99%_) was greater than or equal to 95% of the prescribed dose (60.8Gy) and the mean CTV dose was within 0.5Gy of the prescribed dose. A number of dosimetry parameters were reported using the recommendations of the ICRU Report 83 and ASTRO to compare the treatment plans [18,19]. All plans were evaluated to ensure they met our institutional dose constraints outlined in Table 1. All 3DCRT plans were composed of a 5 beam field arrangement utilising 18MV photons. MLC beam shaping and beam modifiers (eg wedges) were employed as required to produce the most conformal dosimetry. All IMRT and VMAT plans were created using a 6MV photon beam applicable to a Varian Clinac iX (*Varian Medical Systems, Palo Alto, CA*) linear accelerator with a 120 leaf Millennium dynamic multileaf collimator (MLC). 6MV photons were utilised for IMRT and VMAT as this reflected most published clinical studies as well as current practice in our institution.

### Simultaneous infield boost (SIB)

10 of the 15 patients whom had well localised tumours were considered for a SIB. Patients with multiple tumours were excluded from SIB planning. For the SIB the bladder tumour/site of disease was contoured as CTV2 and a 2 cm margin used to create PTV2. The SIB dose was an additional 10Gy, based on a modelling study by [Wright et al. 20], resulting to a total dose of 74Gy in 32 fractions.

### IMRT plans

IMRT plans were generated on the *Eclipse* Version 8.9.08 treatment planning software (*Varian Medical Systems, Palo Alto, CA*) using a 7 beam multifield technique. The initial optimisation parameters and their priorities were set according to our institutional optimisation protocol and then adjusted as required to achieve the dose constraints (Table 1) using a minimum of 60 iterations. A Normal Tissue Objective was included and the default smoothing parameters were applied to help reduce hotspots outside the PTV and the total monitor units.

### VMAT plans

VMAT plans utilising the Varian RapidArc technique (*Varian Medical Systems, Palo Alto, CA*) were planned using *Eclipse* Version 8.9.08 treatment planning software using the same CT-dataset and contoured volumes as the IMRT plans. A single arc technique was used with the gantry set to rotate through 340° in a clockwise direction from a starting position of 190° to a final position of 170° to reduce the amount of treatment through the rectum and central rail of the couch top. The collimator rotation was individually optimised for each patient but generally set at 45° to reduce the effect of tongue and groove leakage.

The final dose calculation for all 3 of the plans for each patient was performed using the anisotropic analytical algorithm (AAA) version 8.9.08 with a 2.5mm dose calculation grid space.

### Delivery times

Treatment was delivered for each of the 15 3D-CRT, IMRT and VMAT plans using a Varian 21iX Linear Accelerator (*Varian Medical Systems, Palo Alto, CA*). All beams were delivered to an empty bunker. Beam delivery time was measured using a stopwatch from the beginning of the first beam to the end of the last beam including all gantry movements. Beam order was optimised to simulate live treatment scheduling. As no patient was involved in this process, these treatment times do not include patient set-up time, which would be expected to be the same for all three plans.

### Conformality and homogeneity

The degree of conformality of the plans was measured by the conformity index (*CI*) which is a ratio of the volume of tissue receiving at least 95% of the prescribed dose divided by the volume of the PTV (Equation 1). A CI value closer to 1 is more conformal.

(1) $$CI=\frac{V_{95\%}}{V_{\mathrm{PTV}}}$$

The homogeneity index (*HI*) was also calculated and is the difference between the near-maximum and near-minimum dose normalised to the median dose (Equation 2) and measures the dose homogeneity across the PTV. A *HI* value approaching zero indicates a more homogenous dose distribution within the PTV.

(2) $$HI=\frac{D_{2\%}-D_{98\%}}{D_{50\%}}$$

The dose was normalised to a selected percentage to give 95% of the dose to 99% of the PT. Once each plan was normalised to ensure the PTV dose reflected the prescribed dose, the DVH data was exported as a CSV file with a resolution of 0.1Gy via the export functions embedded in Eclipse for importing into the TCP/NTCP software.

As the definition of CI does not take into account the location and the shape of the 95% isodose volume (V95) relative to the PTV, we also calculated the Paddick’s index which takes into account the coverage of the target volume with the 95% isodose. The Paddick's definition of conformity index is defined as [21],

(3) Paddick Index=TV95^2V95*TV

where TV95 is the target volume (TV) within the 95% isodose volume (V95).

### Radiobiology modelling

Tumour control probability (TCP) and normal tissue complication probabilities (NTCP) were calculated using the equivalent uniform dose (EUD) mathematical models and software code described by [Gay and Niemierko 22]. Briefly, these models determine EUD from the dose volume histogram (DVH) and apply a parameter ‘*a*’ which is specific to the tumour or organ at risk. The parameter *a* can force the EUD to represent maximal, minimal or average dose. In the case of tumours, the *a* parameter has a large negative number so that the EUD for tumours is close to the minimal dose. For normal tissues with serial like architecture, the *a* parameter will have a large positive number, and for normal tissues that exhibit a large volume effect, *a* will have a small positive number. Values for the *a* parameter used in the TCP and NTCP calculations are summarised in Table 2 and were derived from the references shown in this table, or from the values recommended by [Gay and Niemierko 22] if not specified in the relevant reference. The NTCP and TCP models also require values for the α/β ratio, TD_50_ (the dose for 50% complication rate probability) for normal tissues, TCD_50_ (tumour dose to control 50% of tumours irradiated), and γ_50_ (slope of the dose response curve). For γ_50_, we have assumed a value of 4 for late effects and 2 for the PTV unless an alternative value has been suggested by the associated reference. 

**Table 2:** **Values for the*a*parameter used in the TCP and NTCP calculations**

	**TCD**_**50**_	**TD**_**50**_	***a***	**γ**_**50**_	**α/β**	**Reference**
Bladder	59 Gy		−13	2	13	[Wright 20]
Rectal late effects		76.9 Gy	11	4	3	[Michalski 23]
Non-rectal bowel		59 Gy	11	4	3	[Kavanagh 24], [Pan 25]
Left & right femurs		65 Gy	13	2.7	3	Deb [26]

### Statistical analysis

The 3 treatment techniques were compared using Friedman’s test. When the p-value for the test was inferior to 0.05, a pair-wise comparison was conducted to identify which treatment techniques differ using the Wilcoxon test. No correction for multiple testing was used.

## Results

### Dosimetric outcome

Table 3 shows the dosimetric outcomes of the plans created using a 3D-CRT, IMRT or VMAT technique. Using a range of dosimetry parameters, we found no statistically significant difference in target volume coverage or dose to the OARs except for the femurs that achieved a lower dose for the IMRT and VMAT techniques compared with 3DCRT. With SIB the dosimetric parameters for in favour of VMAT, such as for left femur and non-rectal bowel, none of these can be considered a clinical significant difference.

**Table 3:** Dosmetric values for whole bladder treatment

	**3DCRT**	**IMRT**	**VMAT**
	**Mean (min-max, st dev)**	**Mean (min-max, stdev)**	**Mean (min-max, stdev)**
CTV MAX	66.8 (65.1-68.4, 0.94)	66.1 (65.5-66.7, 0.41)	66.7(65.7-68, 0.65)
CTV MIN	63.1 (62.2-63.7,0.44)	62.4 (61.9-63.1, 0.34)	61.8(61–62.6, 0.49)
CTV MEAN	64.4 (64.2-64.5, 0.11)	64.3 (63.8-64.5, 0.2)	64.3(63.9-64.5, 0.19)
CTV MEDIAN	64.2 (63.9-64.4,0.15)	64.4 (63.7-64.6, 0.24)	64.4(63.9-64.6, 0.2)
CTV D98%	63.4 (62.9-63.9,0.3)	63 (62.7-63.7, 0.27)	62.9(62.4-63.5, 0.35)
CTV D2%	66.1 (64.8-67.5,0.74)	65.6 (64.8-66.2, 0.4)	65.6 (64.9-66.1, 0.36)
CTV D99 > 60.8Gy	63.3 (62.8-63.8,0.32)	62.9 (62.6-63.6, 0.28)	62.7(62.2-63.4, 0.39)
PTV MAX	66.9 (65.3-68.5, 0.9)	67 (66–68, 0.63)	68.2(67.1-69.5, 0.7)
PTV MIN	58.9 (56.7-60.6,1,17)	54 (46.5-58.1, 3.68)	54.4(45.2-58.6, 3.16)
PTV MEAN	64.2 (63.9-64.4,0.16)	64.2 (63.9-64.6, 0.2)	64.2(63.5-64.7, 0.33)
PTV MEDIAN	64.2 (63.9-64.4, 0.17)	64.4 (64–64.8, 0.22)	64.3(63.9-64.9, 0.27)
PTV V95% (cc)	624 (299–987, 208)	495 (244–765, 159)	490(246–751, 150)
PTV D95%	62.3 (61.6-63, 0.42)	62.3 (61.5-62.7, 0.31)	61.8(59.6-62.8, 0.82)
PTV D2%	66.5 (65.1-68, 0.82)	65.9 (65.2-66.2, 0.32)	66.3(65.7-67, 0.39)
PTV D50%	64.2 (63.9-64.4, 0.17)	64.4 (64–64.8, 0.22)	64.3(63.9-64.9, 0.28)
PTV D99 > 60.8Gy	61.1 (60.4-62.3, 0.54)	60.4 (59.6-61.3, 0.55)	60.2(57–61.6, 1.11)
PTV D98 > 60.8Gy	61.6 (60.9-62.6,0.51)	61.2 (60.4-61.8, 0.36)	60.8(58.2-62, 0.97)
Paddix Index	0.75 (0.71-0.8, 0.02)	0.92 (0.87-0.97, 0.03)	0.91(0.88-0.94, 0.02)
RECTUM MEDIAN	35 (16.4-58.1,12)	36.7 (19.4-44.8, 5.89)	39.6(19.9-51.3, 7.6)
RECTUM D98%	9.06(1.58-24.4, 8.06)	6.38 (2.19-16.4, 4.93)	7.37(2.54-19.9, 5.3)
RECTUM D2%	62.1(41.6-64.8, 5.71)	62.4 (50.3-65.3, 3.44)	63.3(45–66.8, 5.18)
RECTUM D60 < 40Gy	31.2 (14.2-54.2, 11.2)	33 (14.5-37.9, 5.75)	34.3(14.4-44.9, 8)
RECTUM D50 < 50Gy	35 (16.4-58.2,12)	36.8 (19.4-44.8, 5.85)	39.4(19.9-51.2, 7.71)
RECTUM D40 < 64Gy	40.7 (18.7-60.5, 12)	40.7 (25.1-51.4, 5.77)	44.1(23.4-57.3, 7.96)
RT FEMUR MEDIAN	40 (36–43.2, 2.8)	21.5 (19.5-23, 1.08)	17.3(13.1-21.5, 2.77)
RT FEMUR D98%	9.55 (1.84-31.8, 7.84)	2.62 (1.46-6.61, 1.37)	2.27(1.32-4.2, 0.84)
RT FEMUR D2%	49.9 (41.2-61, 4.9)	34.8 (25.4-51.9, 6.74)	35.1(28–52, 5.99)
RT FEMUR D100 < 35Gy	5.24 (1.32-19, 4.73)	1.74 (1.16-3.2, 0.58)	1.65(0.9-2.83, 0.61)
RT FEMUR D60 < 45Gy	38.5(32.6-42.3, 2.97)	20.6 (18.5-22.2, 1.24)	15.4(11.5-20.5, 3.14)
RT FEMUR D10 < 50Gy	46.4(39.6-50.7, 3.41)	28.6 (23.8-37.5, 3.88)	29(22.6-40.8, 4.23)
LT FEMUR MEDIAN	39.9 (35.4-43.2, 2.83)	22.1(20.7-24.3, 1.07)	15.5(12.1-18.8, 2.4)
LT FEMUR D98%	10.4 (1.6-32.1, 8.66)	2.79 (1.32-7.38, 1.71)	2.51(1.16-5.12, 1.12)
LT FEMUR D2%	47.8 (41.7-51.6, 3.36)	32.3 (26.6-42.6, 4.24)	32.4(26.1-38.9, 3.38)
LT FEMUR D100 < 35Gy	5.45 (1.17-15.8, 4.37)	1.75(0.92-3.28, 0.69)	1.84(0.88-3.88, 0.85)
LT FEMUR D60 < 45Gy	37.3 (29.2-42.1, 3.84)	21.4(19.8-23.4,1.09)	12.9(8.48-15.4, 2.2)
LT FEMUR D10 < 50Gy	45.1(39.4-49.8, 3.38)	26.7(24.5-31.5,1.96)	27.2(21.2-32.2, 2.86)
NON-RECTAL BOWEL MEDIAN	48.9 (20.6-63.2, 14.2)	42.2 (11.3-62.9,14.5)	42(10.5-63, 14.3)
NON-RECTAL BOWEL D98%	13.6 (2.08-21.8, 6.51)	13.1(2.72-30.6,7.94)	12.5(2.73-29.1, 7.63)
NON-RECTAL BOWEL D2%	65 (62.1-67, 1.04)	65.3(64.6-66.1,0.47)	65.6(64.6-66.6, 0.52)
NON-RECTAL BOWEL D30 < 40Gy	58.7 (40.4-64.2, 7.06)	55.6(34.2-64.9,9.65)	55.6(36.5-64.5, 8.94)
NON-RECTAL BOWEL D0 < 45Gy	65.9 (64.3-67.8, 0.88)	66.1(65.3-67,0.54)	66.9(65.7-67.8, 0.64)
SMALL BOWEL MEDIAN	44.4 (16.1-63.5, 17)	38(15.9-63.7,15.2)	35.8(14.7-63.1, 15.6)
SMALL BOWEL D98%	18.1(2.19-55.2, 14.1)	13.4(3.04-29.4, 8.53)	12.2(3.13-27.2, 7.57)
SMALL BOWEL D2%	59.3(39–67.3, 10)	56.5(30–66.1,14.1)	56.3(29.4-66.9, 14.8)
SMALL BOWEL D30 < 40Gy	52.7 (21.3-64.7, 16.6)	49.9(23.6-63.7, 16)	49.5(21.5-64.5, 17.3)
SMALL BOWEL D0 < 45Gy	62.9 (49.9-67.8, 5.66)	60.3(33.5-67, 11.5)	60.4(31.5-68.4, 12.8)
LARGE BOWEL MEDIAN	48.8 (9.27-62.7, 14.9)	42.5(7.71-64, 15.1)	42.8(6.58-64, 14.7)
LARGE BOWEL D98%	13.5 (1.9-20.6, 6.11)	12.9(2.34-30.6, 7.62)	12(2.26-29.1, 7.26)
LARGE BOWEL D2%	64.9 (63.3-66.5, 0.92)	65.1(64.3-65.8, 0.48)	65.3(64.1-66.2, 0.65)
LARGE BOWEL D30 < 40Gy	58.9 (36.4-64, 7.24)	55.1(32.1-64.7, 9.77)	54.4(25.9-64.9, 11.3)
LARGE BOWEL D0 < 45Gy	65.5 (62.9-67.5, 1.12)	66(65.3-66.9, 0.48)	66.7(65.7-68.4, 0.77)

### PTV conformity index (CI), homogenity index (HI), and Paddick’s index

The mean PTV conformity (CI) index for 3D-CRT was 1.32, for IMRT 1.05, and for VMAT 1.05. The PTV HI index was 0.080 for 3D-CRT, 0.073 for IMRT and 0.086 for VMAT. The Paddick index was smaller for 3D-CRT than IMRT (−0.18, p < 0.001), and VMAT (−0.16, p < 0.001). The Paddick index difference between IMRT and VMAT (0.02, p < 0.015) is considered clinically insignificant.

### Planning time

The total planning time was found to be a mean of 30.7 minutes (range 10–45 minutes) for 3D-CRT; 49.33 minutes (range 20–90 minutes) for IMRT; and 141 minutes (60–240 minutes) for VMAT. Total times on a networked Eclipse system would be shorter, since calculations are distributed across multiple workstations when using AAA algorithm. 3DCRT was significantly quicker than VMAT and IMRT (P < 0.05) while IMRT was quicker than VMAT (P < 0.05). There was no obvious trend in planning times (such as a decrease in time with experience) over the study period.

### Monitor units

The mean monitor units was lowest for 3D-CRT at 267 (range 250–293); was 403 (range 333–489) for VMAT, and highest at 824 (range 641–1083) for IMRT; (P < 0.05).

### Treatment times

Average treatment delivery time were 2:25 min (range 2:01–3:09) for 3D-CRT; 4:39 min (range 3:41–6:40) for IMRT; and were shortest at 1:14 min (range 1:13–1:14) for VMAT.

### Tumour control probability and normal tissue complication probabilities

Table 4 shows the TCP and NTCP for 3D-CRT, IMRT and VMAT. As can be seen most probabilities are similar. With the use of IMRT and VMAT the probability of femoral head complications was lower that with 3D-CRT.

**Table 4:** Tumour Control and Normal Tissue complication Probabilities

	**3D-CRT**	**IMRT**	**VMAT**
	**Mean(min-max, st dev)**	**Mean(min-max, st dev)**	**Mean(min-max, st dev)**
EUD PTV	64(63.7-64.3, 0.19)	64(63.7-64.5, 0.23)	63.9(62.6-64.5, 0.49)
TCP PTV	66%(65%-67%, 1%)	66%(65%-67%, 1%)	65%(62%-67%, 1%)
EUD rectum	52.9(36.1-58, 5.04)	51.1(36.8-57.7, 4.63)	52.5(37.2-59.2, 4.94)
EUD RTFEM	38.9(31.2-47.8, 4.29)	24.9(16.6-41.8, 6.51)	24.3(18.4-40.3, 5.88)
EUD NRB	57.8(49.4-62.1, 3.18)	57.2(50.2-61.6, 2.93)	57.3(50.7-61.6, 2.95)
NTCP NRB	44%(5%-69%, 18%)	40%(7%-67%, 17%)	40%(8%-67%, 17%)
EUD LFH	37.1(31–41.2, 3.23)	22.9(17.6-29.2, 3.46)	21.7(16.6-28, 2.99)

### Simultaneous infield boost (SIB)

Table 5 shows the comparison of IMRT and VMAT for a SIB in the 10 patients with well demarcated tumours. VMAT was associated with a longer planning time but reduced MUs for treatment. Doses to rectum, small bowel and non rectal bowel were comparable for 3D-CRT without boost, versus IMRT and VMAT with SIB. This is illustrated in Table 5.

**Table 5:** SIB comparison using IMRT and VMAT

**Variable**	**IMRT BOOST**	**VMAT BOOST**	
	**Mean(min-max, st dev)**	**Mean(min-max, st dev)**	**p-value**
CTV MAX	76.1(75.1-77.2, 0.69)	77.4(76–78.4, 0.66)	0.004
CTV MIN	63.2(61.7-66.3, 1.33)	61.5(60.2-63.5, 1)	0.011
CTV MEAN	71.5(68.7- 74.3, 1.75)	71.2(68.6-73.2, 1.43)	0.398
CTV MEDIAN	72.3(67–74.6, 2.88)	71.9(66.9-74.1, 2.62)	0.068
CTV D98%	64.8(62.8-69.7, 2.2)	64.4(62.9-68, 1.73)	0.625
CTV D2%	75.4(74.6-76.4-0.55)	76(75–76.6, 0.48)	0.007
CTV D99 > 60.8Gy	64.5(62.5-69.1, 2.08)	63.8(62.4-69.1, 1.85)	0.044
PTV MAX	76.7(75.1-78.3, 0.9)	78.3(76.1-80.2, 1.15)	0.006
PTV MIN	55.1(47.3-57.4, 2.9)	55.5(15.5-57.6, 1.93)	0.476
PTV MEAN	68.8(67.5-70.4, 1.2)	68.8(67–70.4, 0.89)	1.000
PTV MEDIAN	67.9(65.5-71.6, 2.58)	67.7(65.9. 70.1, 1.37)	0.688
PTV V95% (cc)	557(254–798, 171)	552(267–804, 173)	0.756
PTV D95%	63.1(62.5-63.7, 0.46)	62.9(62–63.9, 0.56)	0.450
PTV D2%	75.5(74.5-76.3, 0.49)	75.9(73.4-76.8, 0.96)	0.056
PTV D50%	68.2(65.8-71.6, 2.45)	67.7(65.9-70.1, 1.37)	0.350
PTV D99 > 60.8Gy	61.5(60.5-62.5, 0.58)	61.9(60.3-70, 2.73)	0.307
PTV D98 > 60.8Gy	62.2(61.5-62.9, 0.47)	61.9(61–63, 0.63)	0.307
BOOST GTV MAX	75.7(74.9-76.9, 0.57)	76.6(75.4-77.6, 0.74)	0.006
BOOST GTV MIN	73.2(72.3-74, 0.52)	72.9(71–77.2, 1.58)	0.142
BOOST GTV MEAN	74.5(74.4-74.5, 0.02)	74.5(74.4-74.5, 0.03)	0.357
BOOST GTV MEDIAN	74.5(74.2-74.6, 0.1)	74.5(74.4-74.6, 0.04)	0.308
BOOST GTV D98%	73.5(72.9-74.2, 0.45)	73.2(72.5-73.8, 0.39)	0.075
BOOST GTV D2%	75.3(74.8-76, 0.38)	75.7(75.1-76.5, 0.46)	0.029
BOOST GTV D99 > 70.30Gy	73.5(72.7-74.2, 0.49)	73.1(72.2-73.3, 0.46)	0.068
BOOST PTV MAX	76.6(75.1-78.3, 0.9)	78.6(77–80.2, 0.9)	0.004
BOOST PTV MIN	66.4(63.3-69.5, 1.9)	67.1(65.2-69.5, 1.45)	0.168
BOOST PTV MEAN	74.2(73.6-74.5, 0.26)	74.1(73.7-74.4, 0.22)	0.056
BOOST PTV MEDIAN	74.4(73.8-74.6, 0.28)	74.3(74–74.6, 0.17)	0.350
BOOST PTV V95% (cc)	193(60.8-273, 65.1)	179(61.3-250, 55.9)	0.083
BOOST PTV D95%	71.9(71–72.9, 0.65)	71.3(70.2-72.2, 0.6)	0.023
BOOST PTV D2%	75.8(74.7-76.6, 0.53)	76.7(75.8-77.5, 0.48)	0.004
BOOST PTV D50%	74.4(73.8-74.6, 0.28)	74.3(74–74.6, 0.17)	0.350
BOOST PTV D99 > 70.30Gy	70.4(68–72, 1.08)	70.3(69.3-72, 0.78)	0.683
BOOST PTV D98 > 70.30Gy	71.1(69.9-72.4, 0.81)	70.7(69.7-72.4, 0.75)	0.185
RECTUM MEDIAN	36.4(19.2-43.9, 6.31)	37.1(19.2-49.1, 8.02)	0.351
RECTUM D98%	5.36(2.28-15.4, 4.3)	6.3(2.62-19.5, 4.78)	0.045
RECTUM D2%	66.1(50.8-72.6, 6.18)	66.8(44–75, 8.44)	0.100
RECTUM D60 < 40Gy	32.5(14.5-38, 6.39)	32.5(14.9-41.9, 7.6)	0.423
RECTUM D50 < 50Gy	36.4(19.2-43.9, 6.32)	37.1(19.2-49.1, 8)	0.415
RECTUM D40 < 64Gy	41(24–52.5, 6.9)	41.8(22.7-55.9, 8.68)	0.398
RT FEMUR MEDIAN	24(19.5-27.9, 2.23)	23.9(16.6-31.8, 4.28)	0.965
RT FEMUR D98%	3.07(1.73-6.82, 1.54)	2.86(1.18-5.45, 1.09)	0.625
RT FEMUR D2%	39.5(30.7-54.8, 7.3)	42.4(33–55, 5.96)	0.142
RT FEMUR D100 < 35Gy	1.96(1.17-3.33, 0.65)	2.1(1.28-3.96, 0.75)	0.126
RT FEMUR D60 < 45Gy	22.9(17.5-27.2, 2.59)	22.8(14.4-34.5, 5.6)	0.760
RT FEMUR D10 < 50Gy	31.8(27.1-39, 3.83)	36.8(29.9-47.4, 4.86)	0.041
LT FEMUR MEDIAN	24(19.2-28, 2.27)	20(16.2-24.6, 2.31)	0.009
LT FEMUR D98%	3.18(1.73-7.74, 1.87)	3.19(1.81-5.81, 1.35)	0.213
LT FEMUR D2%	35.5(29.6-46.9, 5.33)	37.4(30.1-45.5, 5.57)	0.505
LT FEMUR D100 < 35Gy	1.96(1.2-3.33, 0.62)	2.27(1.28-3.96, 0.91)	0.044
LT FEMUR D60 < 45Gy	23.1(17.9-27.1, 2.35)	18.5(14.8-24.1, 2.66)	0.008
LT FEMUR D10 < 50Gy	29.2(27–32.9, 2.13)	32.7(27.4-40.2, 4.7)	0.053
NON-RECTAL BOWEL MEDIAN	48.3(29.5-65.8, 11.6)	46.3(28.7-63.6, 12.1)	0.029
NON-RECTAL BOWEL D98%	15.6(7.5-26.9, 7.38)	13.5(6.63-31.9, 7.86)	0.045
NON-RECTAL BOWEL D2%	72.3(66.8-75.5, 3.13)	72.3(67–76.1, 3.37)	0.894
NON-RECTAL BOWEL D30 < 40Gy	59.2(33.6-71.7, 9.99)	58.4(37.2-68.8, 9.38)	0.351
NON-RECTAL BOWEL D0 < 45Gy	75.3(71.5-77.1, 1.53)	76.2(71.6-78.7, 2.19)	0.023
SMALL BOWEL MEDIAN	42.5(18.3-65.7, 14.8)	37.5(14.4-63.5, 16.6)	0.019
SMALL BOWEL D98%	16.7(5.81-33.6, 9.95)	12.7(5.88-25.7, 6.73)	0.008
SMALL BOWEL D2%	62(37–75.8, 16.4)	59.5(28–77.2, 20.4)	0.221
SMALL BOWEL D30 < 40Gy	50.9(23.3-70.5, 17.1)	47.2(17.3-67.2, 20.4)	0.076
SMALL BOWEL D0 < 45Gy	68.8(43.5-76.3, 13)	67.6(30–78.7, 17.5)	1.000
LARGE BOWEL MEDIAN	50.7(28.4-67, 11.1)	48.5(25.4-65, 12.2)	0.023
LARGE BOWEL D98%	15.1(6.46-26.9, 6.71)	13.6(6.23-31.9, 7.34)	0.068
LARGE BOWEL D2%	71.3(65.3-75.7, 3.29)	71.6(66.8-76.3, 3.19)	0.286
LARGE BOWEL D30 < 40Gy	61.7(45.5-73.3, 6.52)	60.2(37.8-72.9, 8.91)	0.168
LARGE BOWEL D0 < 45Gy	74.8(71.5-77.1, 1.52)	75.4(71.6-78.6, 2.3)	0.142
PLANNING TIME (MINS)	65.5(45–100, 19.7)	159(60–250, 54.3)	0.005
TOTAL MU	822(684–1047, 94.1)	419(359–464, 34.4)	0.004
PTV CONFORMITY INDEX (CI)	1.12(1.04-1.29, 0.07)	1.11(1.05-1.22, 0.05)	0.953
PTV BOOST CONFORMITY INDEX (CI)	1.14(0.99-1.48, 0.16)	1.06(0.97-1.23, 0.08)	0.068
PTV HOMOGENITY INDEX (HI)	0.196(0.18-0.22, 0.014)	0.2(0.08-0.24, 0.04)	0.258
PTV BOOST HOMOGENITY INDEX (HI)	0.06(0.03-0.08, 0.02)	0.081(0.05-0.1, 0.014)	0.009
EUD PTV	67.3(65.8-69, 1.24)	67.3 (66–69.1, 0.8)	0.965
TCP PTV	74%(71%-78%, 3%)	74%(71%-78%, 2%)	0.965
EUD PTV2	66(64.6-67.7, 1.21)	66(64.7-67.7, 0.78)	0.965
TCP PTV2	71%(67%-75%, 3%)	71%(68%-75%, 2%)	0.965
EUD rectum	54.1(37.1-61.6, 6.71)	55.6(38.7-64.4, 7.11)	0.011
EUD RTFEM	29.4(21.1-43.7, 6.42)	30.6(22.1- 42.9, 5.8)	0.505
EUD NRB	62.6(52.4-72.1, 5.05)	62.6(52.8-71, 4.95)	0.894
NTCP NRB	68%(13%-96%, 23%)	67%(14%-95%, 23%	0.722
EUD LFH	24.5(19.8-32.4, 3.81)	25.9(19.6-33.2, 4.89)	0.450

## Discussion

Minimising time taken in the treatment room is important for a variety of reasons including patient satisfaction, as well as departmental throughput. In addition, the bladder is a dynamic soft tissue organ, the size and shape can vary according to urine filling and its position can vary according to its size as well as due to rectal filling. Hence it would seem to be the ideal organ for daily online imaging with corrections based on soft tissue position [16,27,28]. The planning datasets in this study are all from patients treated using daily online image guided adaptive radiotherapy [16]. Intrafraction motion and bladder filling is a concern if there is a significant daily treatment duration. Keeping the treatment time as short as possible is important to reduce intrafraction filling as a recent MRI based study has shown increased filling in intervals up to 28 minutes [5]. Studies using ultrasound [29] as well as MRI [30] have shown in certain cases filling and motion during time in the treatment bunker can be significant. With changes in technology such as faster CBCT reconstruction times, increased treatment automation and volumetric arc therapy, time in the treatment bunker is reducing.

A number of studies in other tumour sites have shown that VMAT results in similar plan quality with substantially reduced treatment times compared to IMRT [7,14,31,32]. The combination of similar plan quality and reduced treatment time shows the clear advantage of VMAT over IMRT. In an early report of IMRT in muscle invasive bladder cancer, [Budgell et al. 33] found that using a 4 field IMRT technique was feasible and took an average of 20 minutes treatment delivery time per treatment fraction. [Muren et al. 34] examined concomitant tumour boost for bladder cancer using IMRT, finding such a technique feasible in 9 of 10 planned cases. It is important to note that in only 50% of cases, the dosimetric criteria were met with a 5 field plan but in 90% of cases the criteria were meet using a 7 or 9 field plan [34] at the penalty of increased low dose irradiation of surrounding normal tissues. While there has been an examination of VMAT for simultaneous integrated boost for intraprostatic lesions [35] there is little reported in the literature for bladder cancer.

In our study all contouring and planning was conducted by the same investigators prospectively. It may be possible that the longer VMAT planning times may be reduced as the staff become even more experienced in this newer technology. However we did not find a trend of planning times decreasing over the study period.

While our study did not specifically examine radiation-induced second malignancies, they are an infrequent but feared complication of treatment [36]. IMRT leads to an increase in monitor units compared to 3D-CRT and therefore is likely to increase the integral dose [6]. Ruben et al. found that the effect on secondary cancer induction by spreading out the low to intermediate dose with IMRT is small [37]. While IMRT increases the MU demand compared to 3D-CRT, the smaller field size and reduced average filed intensity have been reported to reduce the scatter more than sufficiently to compensate for any increase in head leakage [37]. The decrease in MUs required with VMAT reduces exposure to leaked radiation from the gantry head, which is a concern regarding the development of second cancers [38]. However VMAT delivers dose circumferentially around patients, potentially leading to an increase in the volume of tissue exposed to low radiation doses. While we found that the monitor units with VMAT was significantly lower than for IMRT, as both the delivered dose distribution and leakage radiation play a role in depositing dose outside the treatment volume, the potential for secondary malignancies after curative treatment remains uncertain across these two techniques [10].

Reduced MUs does have other advantages in the running of radiotherapy departments including extended linear accelerator lifespan, reduced shielding requirements as well as the likely economic benefit of faster treatment and throughput. The reduction in treatment times with use of VMAT are particularly useful for bladder cancer treatment given the organ can fill with urine and move with increasing treatment times. The results of our study for VMAT are promising, but only a randomised clinical study can show the real effects of the introduction of such technique. Unfortunately, with low levels of referral for organ preservation for bladder cancer in many countries, such randomised studies remain unlikely.

## Conclusions

Radiotherapy to the bladder is complicated by the fact that bladder volume may increase during delivery. If bladder cancer patients are to benefit from modern radiotherapy such as IMRT, it is essential to deliver the treatment as fast as possible. We were able to demonstrate that IMRT and VMAT plans are similar in terms of dose distributions and both have superior conformity indices when compared to 3DCRT. Given the benefits in terms of reduced MU as well as treatment time, VMAT appears to be the ideal technology to be used with daily image guided or adaptive radiotherapy for muscle invasive bladder cancer.

## Competing interests

The authors declare that they have no competing interests.

## Authors’ contributions

FF devised the study protocol, carried out all the contouring, wrote the manuscript draft and had overall responsibility for the study. LW carried all the 3D-CRT, IMRT and VMAT plans as well as providing input into the manuscript. MB provided statistical input into the protocol and carried out all the statistical analysis, as well as input into the manuscript. AH advised regarding the study protocol, carried all the radiobiological modelling and provided input into the final manuscript. CH provided radiation therapy input into protocol, advice during the planning process as well as input into the final manuscript. DP provided input into study design. Transferred all datasets for planning, carried out all treatment timings as well as input into the manuscript. JC provided medical physics input into the study design, conduct as well as the manuscript. SC provided input into the study design, protocol text and manuscript text KHT provided input into the study design, protocol text and manuscript text. TK provided overall medical physics input and advice into the protocol, study conduct and manuscript. All authors read and approved the final manuscript.
